# An Extracellular Pore‑Targeting Peptide Defines a Designable Allosteric Site in TRPV2

**DOI:** 10.1002/advs.77759

**Published:** 2026-09-27

**Authors:** Aiqin Zhu, Hongkun Wang, Jiawei Wang, Xiaolu Liu, Aerziguli Aierken, Lizhen Xu, Ping Liang, Fan Yang

**Affiliations:** ^1^ Kidney Disease Center of the First Affiliated Hospital and Department of Biophysics Zhejiang University School of Medicine Hangzhou Zhejiang China; ^2^ Liangzhu Laboratory Zhejiang University Medical Center Zhejiang University School of Medicine Hangzhou Zhejiang China; ^3^ Key Laboratory of combined Multi‐organ Transplantation Ministry of Public Health, the First Affiliated Hospital Zhejiang University School of Medicine Hangzhou Zhejiang China; ^4^ Institute of Translational Medicine Zhejiang University Hangzhou Zhejiang China; ^5^ Hangzhou Institute of Medicine (HIM) Chinese Academy of Sciences Hangzhou Zhejiang China; ^6^ School of Basic Medical Sciences and Forensic Medicine Hangzhou Medical College Hangzhou Zhejiang China; ^7^ Department of Physiology School of Basic Medical Sciences Xinjiang Medical University Urumqi Xinjiang China; ^8^ Xinjiang Key Laboratory of Molecular Biology for Endemic Diseases Urumqi Xinjiang China; ^9^ Scientific Research Platform of Xinjiang Medical University Key Laboratory of Neurophysiology and Clinical Translation Urumqi Xinjiang China

**Keywords:** allosteric regulation, biochemistry, biophysics, chemistry, extracellular, ion channel, peptide binding, peptide, transient receptor potential channel

## Abstract

Extracellular pore domains of ion channels are emerging as dynamic regulatory surfaces, but whether they can be rationally targeted to achieve selective channel modulation remains unclear. Here, using an optimized hotspot‐centric design strategy, we developed Depiv2, a structure‐guided peptidic inhibitor of TRPV2 that binds the extracellular pore domain and suppresses channel activity with nanomolar potency and subtype selectivity. Our cryo‐EM structure of the TRPV2–Depiv2 complex showed that peptide binding remodels the pore domain and stabilizes a closed, non‐conductive conformation. Patch‐clamp recordings further revealed that Depiv2 decreases both open probability and single‐channel conductance, indicating inhibition through combined allosteric and permeation‐coupled mechanisms. In cellular and mouse models of pathological cardiac hypertrophy, Depiv2 blunted disease‐associated remodeling. These findings establish the extracellular pore of TRPV2 as a designable allosteric site and illustrate how rational peptide engineering can be used to target extracellular regulatory surfaces in TRP channels.

## Graphical abstract

Structure‐guided peptide engineering yields Depiv2, a highly potent and subtype‐selective TRPV2 inhibitor that binds to the extracellular pore and remodels it into a closed, non‐conductive state. Depiv2 suppresses pathological cardiac hypertrophy, establishing the TRPV2 outer pore as a designable interface for selective peptide modulation.

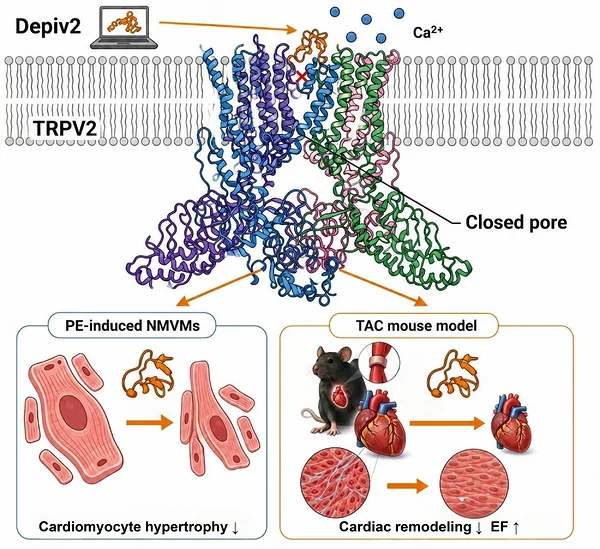

## 1 Introduction

Ion channels are most commonly modulated through orthosteric ligand‐binding pockets, transmembrane allosteric sites, or direct pore blockade [1]. Yet growing evidence suggests that extracellular pore domains can function as dynamic regulatory surfaces rather than passive conduits for ion permeation [2, 3]. Whether such extracellular pore surfaces can be rationally targeted to achieve potent and selective channel modulation remains elusive.

This question is particularly compelling in the transient receptor potential (TRP) channel family, whose members are polymodal sensors with highly dynamic pore domains [4, 5]. In TRPV1, the extracellular pore domain has emerged as a direct determinant of thermal gating: the pore turret forms part of the temperature‐activation pathway [3], and perturbing this region selectively disrupts high‐sensitivity heat activation while sparing capsaicin activation [6]. The centipede toxin RhTx further reinforced this principle by binding the extracellular pore domain of TRPV1 and directly affecting heat activation [7]. Together, these studies establish the extracellular pore domain of TRP channels as a mechanistically privileged component of the gating machinery. Consistent with this view, engineered peptides can also engage extracellular pore‐associated structures in TRPV1 [8] and TRPM8 [9].

The extracellular pore is also attractive from a ligand‐design perspective. Peptides are well suited to broad and chemically heterogeneous protein surfaces, but their use against intracellular targets is often limited by poor membrane permeability. Although cell‐penetrating sequences such as TAT can improve intracellular access [10], they increase molecular complexity and may compromise affinity or selectivity. By contrast, extracellular channel surfaces are directly accessible, and the ion‐channel pore domain is especially favorable because it is solvent‐exposed, structurally organized, and tightly coupled to gating.

TRPV2 provides a stringent test of this idea. This calcium‐permeable TRP channel contributes to mechanosensitive and stress‐responsive signaling [11, 12, 13, 14]. Notably, its expression in multiple cardiac cell types, including aortic myocytes and cardiomyocytes [15, 16], is significantly upregulated in the transverse aortic constriction (TAC) model of pressure overload–induced hypertrophy [16]. Genetic ablation of TRPV2 reduces left ventricular hypertrophy in this model [16]. However, currently available pharmacological tools remain inadequate for precise mechanistic interrogation. Though the TRPV2 inhibitor tranilast has improved cardiac function in patients with advanced heart failure [17, 18], it lacks the potency and selectivity required for optimized modulation. From the mechanistic perspective, though natural toxins, such as the centipede toxin RhTx, have demonstrated that the outer pores of TRP channels can be targeted to modulate gating, these molecules are products of evolutionary selection rather than rational invention. Consequently, it remains elusive whether the extracellular pore domain possesses the sufficient structural and physicochemical determinants to support computational design de novo. Resolving this question would shift the paradigm from the serendipitous discovery of naturally occurring pore‐targeting ligands to the intentional engineering of allosteric modulators. Recent high‐resolution TRPV2 structures therefore create an opportunity to ask whether its extracellular pore domain can be intentionally engaged by a rationally designed peptide ligand [19, 20, 21, 22, 23, 24, 25, 26].

Here we addressed this question using an optimized hotspot‐centric approach (OHCA), which we successfully applied to design peptidic modulators of TRPV1 and TRPM8 [9, 10], to develop peptidic inhibitor of mTRPV2 (Depiv2), a peptidic inhibitor that targets the extracellular pore domain of TRPV2. Guided by structural features of the negatively charged extracellular pore domain, we engineered a compact peptide bearing a complementary positively charged interaction motif that inhibits TRPV2 with nanomolar potency and subtype selectivity. Structural and electrophysiological analyses show that Depiv2 remodels the pore domain, stabilizes a non‐conductive conformation, and inhibits channel function through coupled effects on gating and ion permeation. Together with its ability to suppress pathological remodeling in cellular and mouse models of cardiac hypertrophy, these findings define the extracellular pore of TRPV2 as a designable allosteric site and establish a framework for targeting extracellular regulatory surfaces in TRP channels.

## 2 Results

### 2.1 Rational Design and Functional Characterization of Depiv2

To design peptidic modulators targeting the extracellular pore domain of TRPV2, we applied the OHCA strategy, as described previously [9, 10], which enables peptide design by prioritizing energetically favorable interaction hotspots within target proteins. Based on our previously resolved high‐resolution mouse TRPV2 structure (Protein Data Bank (PDB) ID: 7XEO) [23], we first computationally docked each of the natural amino acids into the extracellular pore domain by Rosetta [27]. We identified the positively charged residue, arginine, as a favorable interaction hotspot within the extracellular pore pocket (Figure S2a–c). As in our previous studies [9, 10], incorporation of this hotspot into structurally compatible scaffolds from the PDB, followed by iterative optimization, yielded a peptidic inhibitor of mTRPV2, termed Designed peptidic inhibitor of mTRPV2 (Depiv2).

**FIGURE 1:**
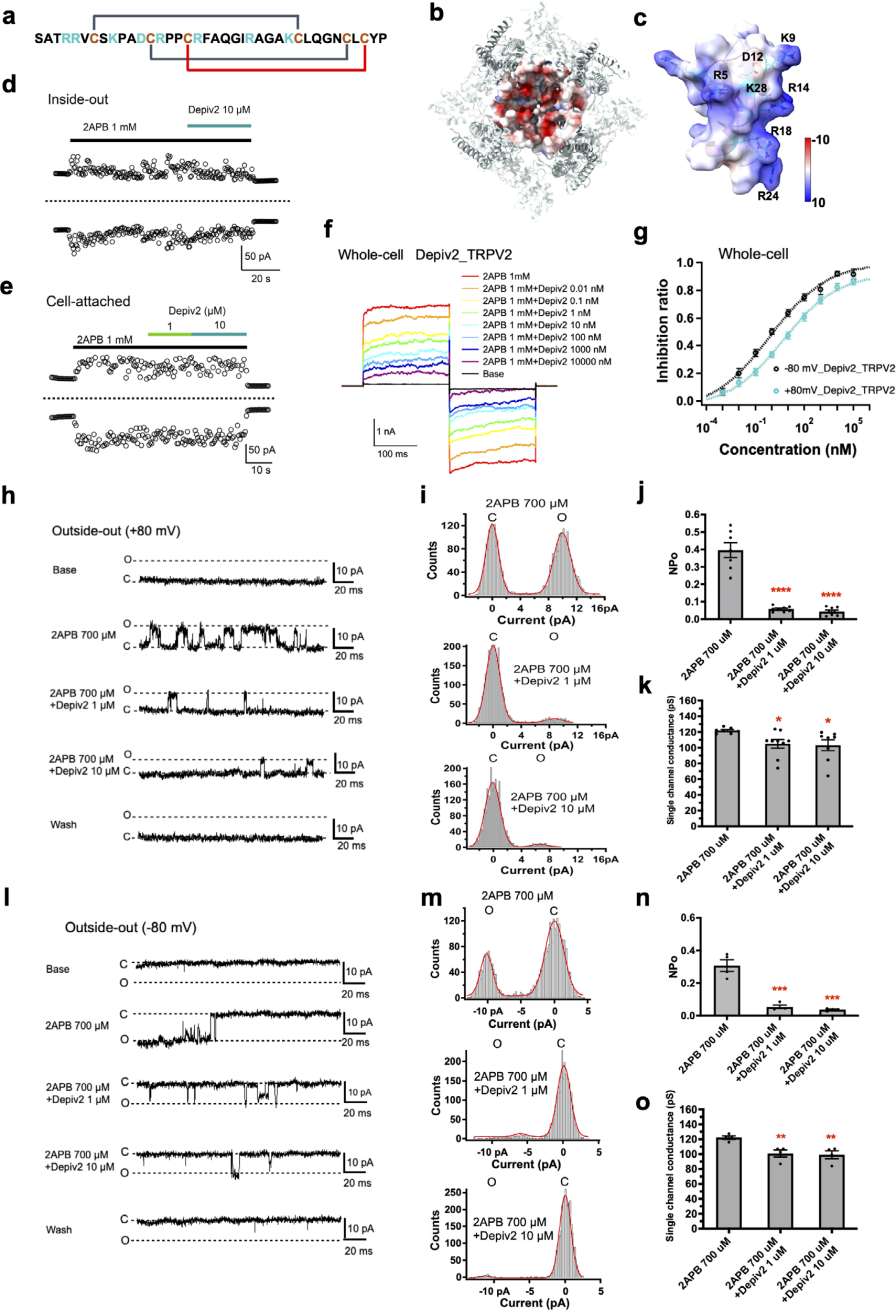
Functional validation of Depiv2. (a) Amino acid sequence of the designed Depiv2. Charged amino acids (highlighted in cyan) and cysteines (highlighted in orange) are marked, and the corresponding disulfide bonds are indicated by straight lines. (b) Top view of mTRPV2 showing the electrostatic potential surface of the extracellular pore domain. (c) Electrostatic potential surface (kcal/mol/e) of the designed peptide is shown in color (red, negative; blue, positive). (d,e) Depiv2 did not inhibit the TRPV2 channel even at concentrations up to 10 µM under cell‐attached (e) or inside‐out (d) recording configurations, recordings were conducted at ±80 mV. (f) Depiv2 inhibited 2‐APB–induced TRPV2 currents in a concentration‐dependent manner, recordings were conducted at ±80 mV. (g) Concentration–response curve of Depiv2 fitted to the Hill equation, yielding IC_50_ of 0.95 ± 0.18 nM (−80 mV) and 5.20 ± 0.56 nM (+80 mV). The inhibition ratio was calculated by measuring the current amplitude inhibited by Depiv2 at different concentrations and normalizing to the current elicited by 2‐APB. Data were collected at −80 mV or +80 mV and are presented as mean ± S.E.M.; *n* = 6 independent cells at 10^−^^3^ nM, *n* = 3 independent cells at 10^5^ nM (*n* = 6 for +80 mV), *n* = 10 independent cells at 0.01 –1 nM and *n* = 6 independent cells per condition for all other concentrations. (h) Representative traces from outside‑out single‑channel recordings of the TRPV2 channel at +80 mV. 2‑APB (700 µM) alone or a mixture of 2‑APB (700 µM) and Depiv2 (1 or 10 µM) was perfused to activate or inhibit the channel, respectively. (i) A double Gaussian function (red) was used to fit the all‑point histogram of the representative single‑channel recordings shown in h. The left Gaussian peak represents the closed state, and the right Gaussian peak represents the open state. (j) *NP_o_* (*N* = 1) of the TRPV2 channel obtained from single‐channel recordings at +80 mV. The *NP_o_* decreased significantly from 39.6 ± 11.3% (700 µM 2‑APB) to 5.8% ± 1.5% (700 µM 2‑APB + 1 µM Depiv2) and 4.4% ± 2.3% (700 µM 2‑APB + 10 µM Depiv2). Data are presented as mean ± S.E.M.; *n* = 7 independent cells for the 2‐APB (700 µM) group, and *n* = 8 independent cells for the 2‐APB (700 µM) + Depiv2 (1 and 10 µM) groups. An unpaired *t*‑test was used to compare differences between groups, ^****^ *p* < 0.0001 compared with “2‐APB 700 µM”. (k) Single‑channel conductance of the TRPV2 channel obtained from single‐channel recordings at +80 mV. The conductance decreased slightly from 121.8 ± 3.5 pS (700 µM 2‑APB) to 104.9 ± 5.4 pS (700 µM 2‑APB + 1 µM Depiv2) and 103.1 ± 6.9 pS (700 µM 2‑APB + 10 µM Depiv2). Data are presented as mean ± S.E.M.; *n* = 7 independent cells for the 700 µM 2‐APB group, *n* = 9 independent cells for 700 µM 2‑APB + 1 µM Depiv2 group, and *n* = 8 independent cells for 700 µM 2‑APB + 10 µM Depiv2 group. An unpaired *t*‑test was used to compare differences between groups; ^*^ *p* < 0.05 compared with “2‐APB 700 µM”. (l) Representative traces from outside‐out single‐channel recordings of the TRPV2 channel at −80 mV. 2‐APB (700 µM) alone or a mixture of 2‐APB (700 µM) and Depiv2 (1 or 10 µM) was perfused to activate or inhibit the channel, respectively. (m) A double Gaussian function (red) was used to fit the all‐point histogram of the representative single‐channel recordings shown in l. The left Gaussian peak represents the open state, and the right Gaussian peak represents the closed state. (n) *NP_o_* (*N* = 1) of the TRPV2 channel obtained from single‐channel recordings at −80 mV. The *NP_o_* decreased from 30.7% ± 3.6% (700 µM 2‐APB) to 5.4% ± 1.0% (700 µM 2‐APB + 1 µM Depiv2) and 3.7% ± 0.4% (700 µM 2‐APB + 10 µM Depiv2). (o) Single‐channel conductance of the TRPV2 channel obtained from single‐channel recordings at −80 mV. The conductance showed a modest reduction from 122.2 ± 2.4 pS (700 µM 2‐APB) to 100.8 ± 4.8 pS (700 µM 2‐APB + 1 µM Depiv2) and 99.1 ± 5.3 pS (700 µM 2‐APB + 10 µM Depiv2). Data are presented as mean ± S.E.M.; *n* = 4 independent cells for each group. Statistical significance was assessed using an unpaired *t*‐test; ^**^ *p* < 0.01, ^***^ *p* < 0.001 compared with the 700 µM 2‐APB group.

Depiv2 comprises 38 amino acids and contains six cysteine residues predicted to form three disulfide bonds (Figure 1a). Electrostatic surface analysis revealed that the extracellular pore domain of TRPV2 is enriched in negatively charged residues (Figure 1b), whereas Depiv2 presents multiple positively charged residues (Figure 1c), supporting favorable electrostatic complementarity.

The linear peptide was first chemically synthesized and then folded in a liquid environment, typically a buffer containing redox pairs. The folded peptide was further purified by reverse‐phase chromatography (RPC) with acetonitrile as the mobile phase. The peak of folded Depiv2 was eluted with 38% acetonitrile (Figure S2d). Mass spectrometry results showed that the molecular mass of folded Depiv2 was 4093 Da, compared with 4099 Da for linear Depiv2, a difference of 6 Da, which was exactly the molecular weight of six H atoms (Figure S2e). Therefore, we concluded that the six cysteines in Depiv2 have formed three disulfide bonds after oxidative folding.

To evaluate its functional activity, we performed patch‐clamp recordings in HEK293T cells expressing mTRPV2. Using 1 mM 2‑APB as the agonist, we first confirmed that the evoked currents were specifically mediated by mTRPV2 across inside‑out, cell‑attached and outside‑out configurations (Figure S5a–c). Depiv2 did not inhibit channel activity in either cell‐attached or inside‐out configurations, even at concentrations up to 10 µM (Figure 1d,e), indicating that it does not act from the intracellular side. In contrast, in whole‐cell recordings, we observed robust inhibition of 2‐APB–induced TRPV2 currents by Depiv2 in a concentration‐dependent manner (Figure 1f and Figure S5d), with an IC_50_ of 0.95 ± 0.18 nM (Figure 1g), demonstrating high potency of Depiv2. Because the holding potential of −80 mV more closely approximates the resting membrane potential, the inhibition ratios presented in the Results section were predominantly derived from recordings obtained at this voltage.

To investigate whether Depiv2 inhibition depends on the mode of TRPV2 activation, we performed additional electrophysiological experiments using alternative TRPV2 agonists, including Probenecid (Figure S6a‐c) and Cannabidiol (CBD) (Figure S6d‐f). Specifically, Depiv2 inhibited Probenecid‑evoked mTRPV2 currents with an IC_50_ of 1.45 ± 0.15 nM (Figure S6k).

We next assessed the inhibitory efficacy of Depiv2 in the presence of 2 mM extracellular Ca^2^^+^. Under these conditions, the IC_50_ for 2‐APB‐evoked mTRPV2 currents was 0.84 ± 0.14 nM (Figure S6g–h,k), comparable to but slightly lower than that obtained in the absence of Ca^2^^+^, possibly reflecting the reduced current amplitude and accelerated desensitization observed in the presence of Ca^2^^+^ (Figure S6c,d). Depiv2 also effectively inhibited CBD‐evoked mTRPV2 currents under these conditions, with an inhibition ratio of 57.14% ± 4.84% at 1 nM Depiv2 (Figure S6e‐f,l). Even at 37°C, 1 nM Depiv2 maintained robust inhibitory activity against mTRPV2 currents, achieving an inhibition ratio of 50.47% ± 4.12%, indicating that Depiv2 retains robust inhibitory activity at a physiologically relevant temperature (Figure S6m–o). We further examined the effect of Depiv2 on human TRPV2 (hTRPV2). In the presence of extracellular Ca^2^^+^, Depiv2 inhibited CBD‐evoked hTRPV2 currents with an IC_50_ of 1.07 ± 0.24 nM (Figure S6i–k), and 1 nM Depiv2 produced an inhibition of 53.22% ± 4.67% (Figure S6l). Collectively, these results suggest that Depiv2 inhibits TRPV2 channel activation by multiple agonists regardless of the presence or absence of calcium ions.

We further performed single‐channel recordings to reveal that Depiv2 markedly reduced channel open probability (*NP_o_*) at both +80 mV (Figure 1h–j and Figure S2i) and −80 mV (Figure 1l–n), while also moderately decreasing single‐channel conductance (Figure 1 k,o), indicating that channel inhibition arises from both altered gating behavior and reduced ion permeation. To assess sequence specificity, we generated a scrambled variant of Depiv2. This control peptide exhibited dramatically reduced potency by more than 5000‐fold (Figure S2f–h), confirming that the native sequence and structural integrity of Depiv2 are essential for effective TRPV2 inhibition.

### 2.2 Depiv2 Exhibits High Subtype Selectivity across Ion Channels

Given that TRPV2 belongs to the TRP channel superfamily [28], whose members share conserved structural architecture, we next assessed the subtype selectivity of Depiv2 across a panel of related ion channels. To further evaluate potential cardiotoxicity, representative voltage‐gated ion channels, including hERG, Nav1.5, and Nav1.7, were also examined.

**FIGURE 2:**
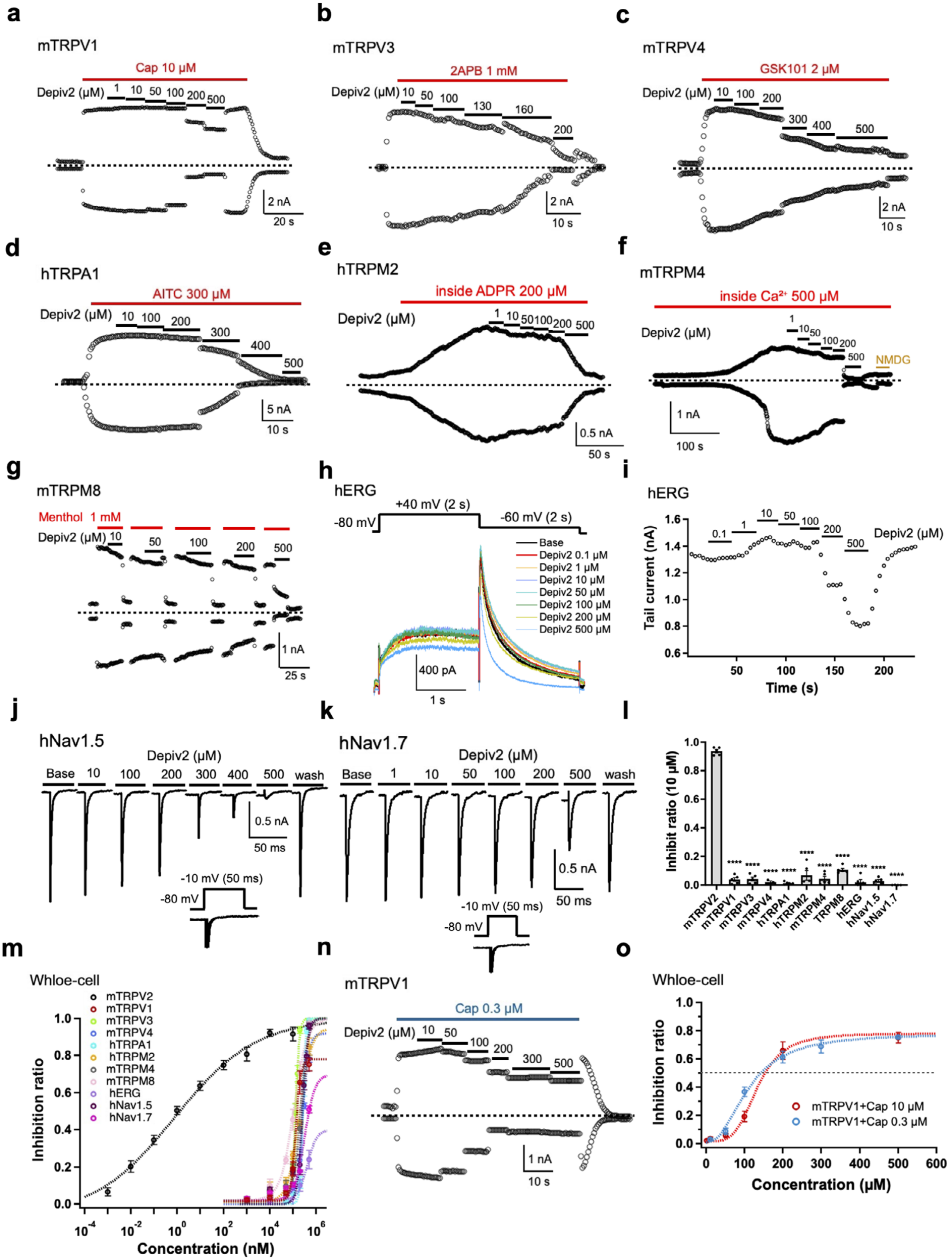
Subtype selectivity of Depiv2. (a–k) Representative whole‐cell patch‐clamp recordings of the indicated ion channels in the presence of Depiv2, including mTRPV1, mTRPV3, mTRPV4, hTRPA1, hTRPM2, mTRPM4 (mTRPM4‐K1045A mutant used to eliminate PIP_2_ dependence), mTRPM8, hERG, hNav1.5 and hNav1.7. hTRPA1, hTRPM2, hERG, hNav1.5 and hNav1.7 are human channels, whereas mTRPV1, mTRPV3, mTRPV4, mTRPM4 and mTRPM8 are mouse channels. Panel **i** shows the averaged tail current of hERG. (l) Inhibition of mTRPV2 compared with other channels at 10 µM Depiv2. The inhibition ratio was calculated by normalizing Depiv2‐inhibited current amplitudes to those elicited by ligand or voltage. TRP currents were measured at ‐80 mV, NaV peak currents at ‐10 mV, hERG tail currents at ‐60 mV, and the data are presented as mean ± S.E.M.; an unpaired two‐tailed Student's *t*‐test was used, *n* = 3–6 independent cells per channel; ^****^ *p* < 0.0001 vs. TRPV2. (m) Inhibition concentration–response curves of Depiv2 on mTRPV2 and other ion channels. Whole‐cell TRP currents were recorded at –80 mV, NaV peak currents at ‐10 mV, hERG tail currents at ‐60 mV, and data are presented as mean ± S.E.M. The concentration–response curve for mTRPV2 (IC_50_ = 0.95 ± 0.18 nM) was derived from Figure 1 g. For mTRPV1 (red), IC_50_ = 134.07 ± 4.18 µM, *n* = 5 independent cells. For mTRPV3 (green), IC_50_ = 132.54 ± 2.65 µM, *n* = 6 independent cells. For mTRPV4 (dark blue), IC_50_ = 281.38 ± 11.10 µM, *n* = 3 independent cells. For hTRPA1 (cyan), IC_50_ = 272.87 ± 4.85 µM, *n* = 6 independent cells. For hTRPM2 (orange), IC_50_ = 194.90 ± 18.80 µM, *n* = 4 independent cells. For mTRPM4 (gray), IC_50_ = 219.34 ± 18.90 µM, *n* = 5 independent cells. For mTRPM8, IC_50_ = 110.55 ± 12.05 µM, *n* = 4 independent cells. For hERG, IC_50_ = 425.05 ± 43.30 µM, *n* = 5 independent cells. For hNav1.5, IC_50_ = 264.97 ± 20.60 µM, *n* = 3 independent cells. For hNav1.7, IC_50_ = 325.47 ± 35.30 µM, *n* = 3 independent cells. (n) Whole‐cell current recordings showing inhibition of capsaicin (0.3 µM)‐activated mTRPV1 currents by Depiv2. (o) Concentration–response curves of Depiv2 inhibition of mTRPV1 currents activated by 0.3 or 10 µM capsaicin. The IC_50_ values were 111.88 ± 4.41 µM and 134.07 ± 4.18 µM for 0.3 and 10 µM capsaicin, respectively.

We performed whole‐cell patch‐clamp recordings and observed that Depiv2 exhibited negligible inhibitory effects on non‐TRPV2 channels, even at micromolar concentrations (Figure 2a–k). Across TRP subtypes, including TRPV1, TRPV3, TRPV4, TRPA1, TRPM2, TRPM4, and TRPM8, channel activity remained largely unaffected. Similarly, we observed no appreciable inhibition for hERG, Nav1.5, or Nav1.7, indicating minimal off‐target effects on voltage‐gated ion channels.

To quantify subtype selectivity, we first assessed the inhibitory effects of 10 µM Depiv2—a saturating concentration that completely suppresses wild‑type TRPV2 currents—across a panel of structurally related and functionally distinct ion channels. At this concentration, Depiv2 showed minimal inhibition of all tested channels (Figure 2l). To further characterize the selectivity profile, we constructed full inhibition concentration–response curves for each channel (Figure 2m). Whole‑cell recordings showed that Depiv2 inhibited mTRPV2 with an IC_50_ of 0.95 ± 0.18 nM, whereas the IC_50_ values for the other channels ranged from 110.55 ± 12.05 to 425.05 ± 43.30 µM. These results demonstrate that Depiv2 exhibits potent and selective inhibition of mTRPV2, with a selectivity window of over five orders of magnitude compared to the other channels tested.

Given the topological and electrostatic similarities between the outer pore of TRPV2 and TRPV1, we further evaluated whether the capsaicin concentration used to activate mTRPV1 influences the inhibitory potency of Depiv2. We therefore compared the concentration‑dependent inhibition of Depiv2 on mTRPV1 currents activated by 0.3 µM vs. 10 µM capsaicin (Figure 2n,o). The IC_50_ value of Depiv2 against mTRPV1 activated by 0.3 µM capsaicin was 111.88 ± 4.41 µM, compared with 134.07 ± 4.18 µM for 10 µM capsaicin. Although the IC_50_ showed a moderate leftward shift of approximately 22 µM at the lower agonist concentration, the overall potency difference remained relatively modest. In both conditions, Depiv2 was substantially less potent against mTRPV1 than against mTRPV2.

### 2.3 Structural Basis for Depiv2‑Mediated Inhibition of TRPV2

**FIGURE 3:**
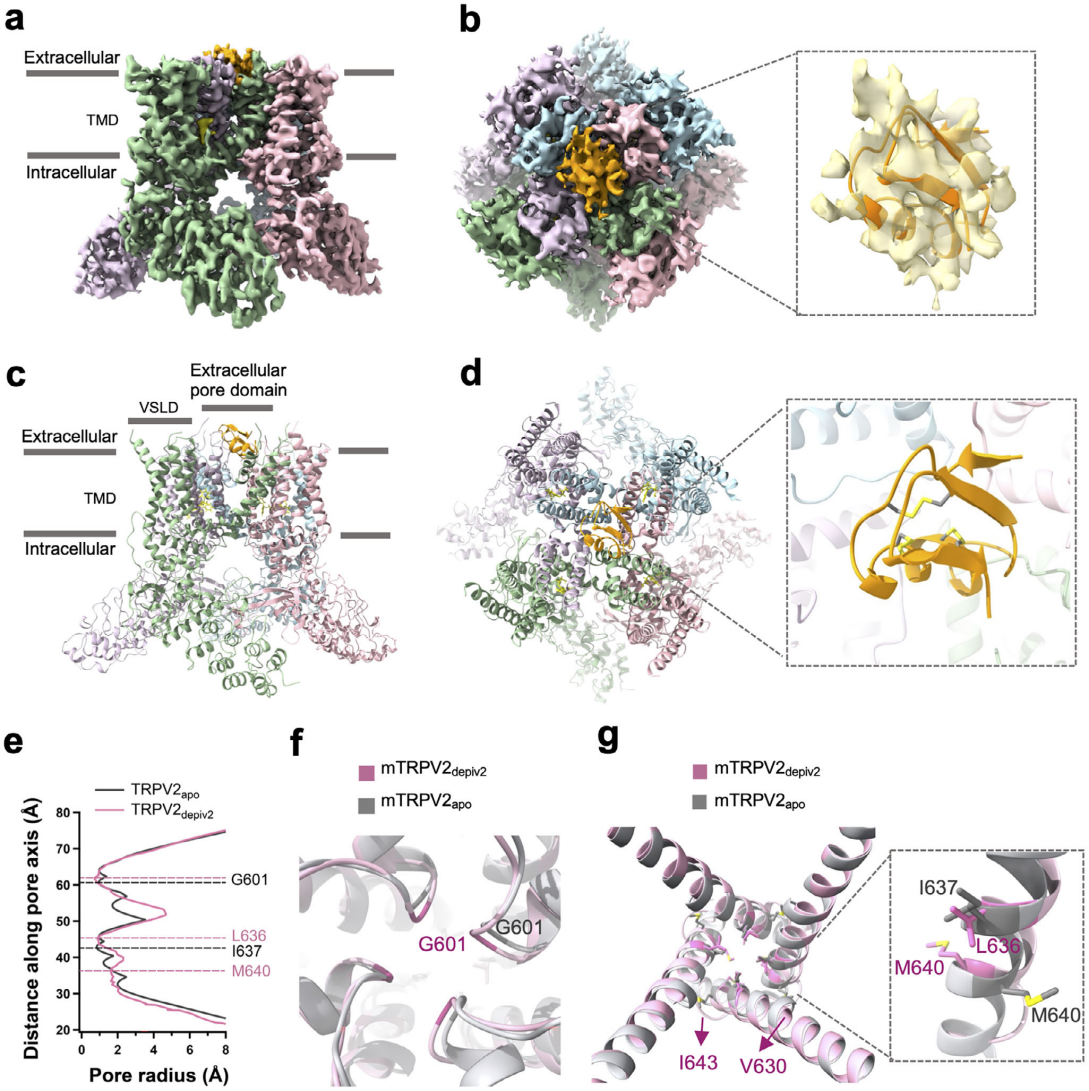
Structural basis of TRPV2 inhibition by Depiv2 binding to the extracellular pore domain. (a,5b) Side (**a**) and top (**b**) views of the cryo‐EM density map of the mTRPV2–Depiv2 complex, with each TRPV2 subunit individually colored and Depiv2 highlighted in orange. Gray bars indicate the approximate position of the cell membrane. An enlarged view of the Depiv2 model fitted into the density is shown on the right. (c,d) Cartoon representations of the side (c) and top (d) views of the mTRPV2–Depiv2 complex, with each TRPV2 subunit individually colored. An enlarged view of the Depiv2 model is shown on the right, with disulfide bonds highlighted in yellow. (e) Pore radius profiles calculated using the HOLE program show constrictions narrower than the radius required for water permeation. (f) Alignment of the pore loops across the four subunits between the mTRPV2–Depiv2 complex and apo mTRPV2. (g) Comparison of the S6 bundle crossing between the mTRPV2–Depiv2 complex and apo mTRPV2.

To elucidate the structural basis of TRPV2 inhibition by Depiv2, we determined the cryo‐electron microscopy (cryo‐EM) structure of the mouse TRPV2–Depiv2 complex at an overall resolution of 3.6 Å, with channel protein reconstituted in nanodiscs and treated with methyl‐β‐cyclodextrin to remove endogenous cholesterol and supplemented with 2‐APB (Figure S1a–g and Table S1) [23]. The overall architecture is consistent with previously reported TRPV2 structures [19, 20, 21, 23, 25, 29], with density attributable to 2‐APB observed at the previously described vanilloid‐binding pocket (VBP) [30], although the molecular assignment of 2‐APB‐associated density remains under discussion (Figure 3a and Figure S1g,h). With C1 symmetry during the entire cryo‐EM data processing, we observed a density attributable to Depiv2 in the extracellular pore domain (Figure 3b,d). Due to the limitation in resolution likely caused by the location of Depiv2 binding, which lies along the symmetry axis of TRPV2, we could not reliably assign side‐chain orientations in Depiv2. Nevertheless, the structure of the Depiv2 backbone resembled our design, with the presence of three β‐strands and one α‐helix forming a compact fold stabilized by three intramolecular disulfide bonds (Figure S3a–c). The Depiv2 binding model was further supported by site‐directed mutagenesis and molecular dynamics simulations.

To further validate the binding configuration of Depiv2 as observed in our cryo‐EM structure, we rebuilt the side chains of Depiv2 and performed all‐atom molecular dynamics simulations. During the 100 ns simulations, Depiv2 remained bound with the pore domain despite moderate conformational flexibility (Figure S3d,e).

To reveal the conformational rearrangements induced by Depiv2 binding, we compared the Depiv2‐bound structure with the apo state (PDB ID: 7XEO), aligning the voltage‐sensor‐like domain (VSLD). Depiv2 binding induces localized rearrangements within the pore domain without altering the overall channel architecture. At the selectivity filter, the pore loop shifts toward the central axis, resulting in a narrowed constriction (Figure 3e,f). More pronounced changes occur at the S6 bundle crossing, where the lower gate adopts a constricted conformation. Specifically, the S6 helix undergoes a *π*‐to‐α transition near residue V630, a structural feature previously associated with channel inactivation [31], leading to a one‐residue register shift and repositioning of the narrowest constriction from I637 in the apo state to L636 in the Depiv2‐bound state (Figure 3e,g). Concurrently, the side chain of M640 rotates toward the pore axis, further restricting the lower gate (Figure 3g). This configuration closely resembles previously reported closed‐state conformations of TRPV2 [19] and related TRPV channels [32, 33, 34], thereby supporting the assignment of a non‐conductive pore architecture. Additionally, the helix near residue I643 undergoes an α‐to‐*π* transition, a structural rearrangement previously observed in TRPV channels that is thought to stabilize the closed pore conformation (Figure 3g) [19, 35]. Consistent with these conformational changes, pore radius analysis shows that both the selectivity filter and lower gate are constricted below the threshold required for ion permeation (Figure 3e).

To assess the state‑dependence of Depiv2 action, we compared its inhibition upon post‑activation application vs. pre‑incubation with Depiv2 in the resting state (Figure S5g,h). Pre‐incubation with 1 nM Depiv2 yielded significantly stronger inhibition (66.09% ± 3.39% vs. 51.25% ± 1.47%), whereas the 2‐APB‐evoked current did not recover to its initial level upon washout. In contrast, in the absence of Depiv2, the 2‐APB‐evoked current fully recovered to its initial level (Figure S5f). These results indicate that Depiv2 preferentially stabilizes the closed state of TRPV2 rather than acting as a simple pore blocker. Collectively, these structural rearrangements and electrophysiological recordings provide a mechanistic basis for the observed inhibition, in which Depiv2 reduces both channel open probability (Figure 1j,n) and single‐channel conductance (Figure 1k,o), consistent with stabilization of a closed state of TRPV2.

### 2.4 Functional Mechanism of TRPV2 Inhibition by Depiv2

**FIGURE 4:**
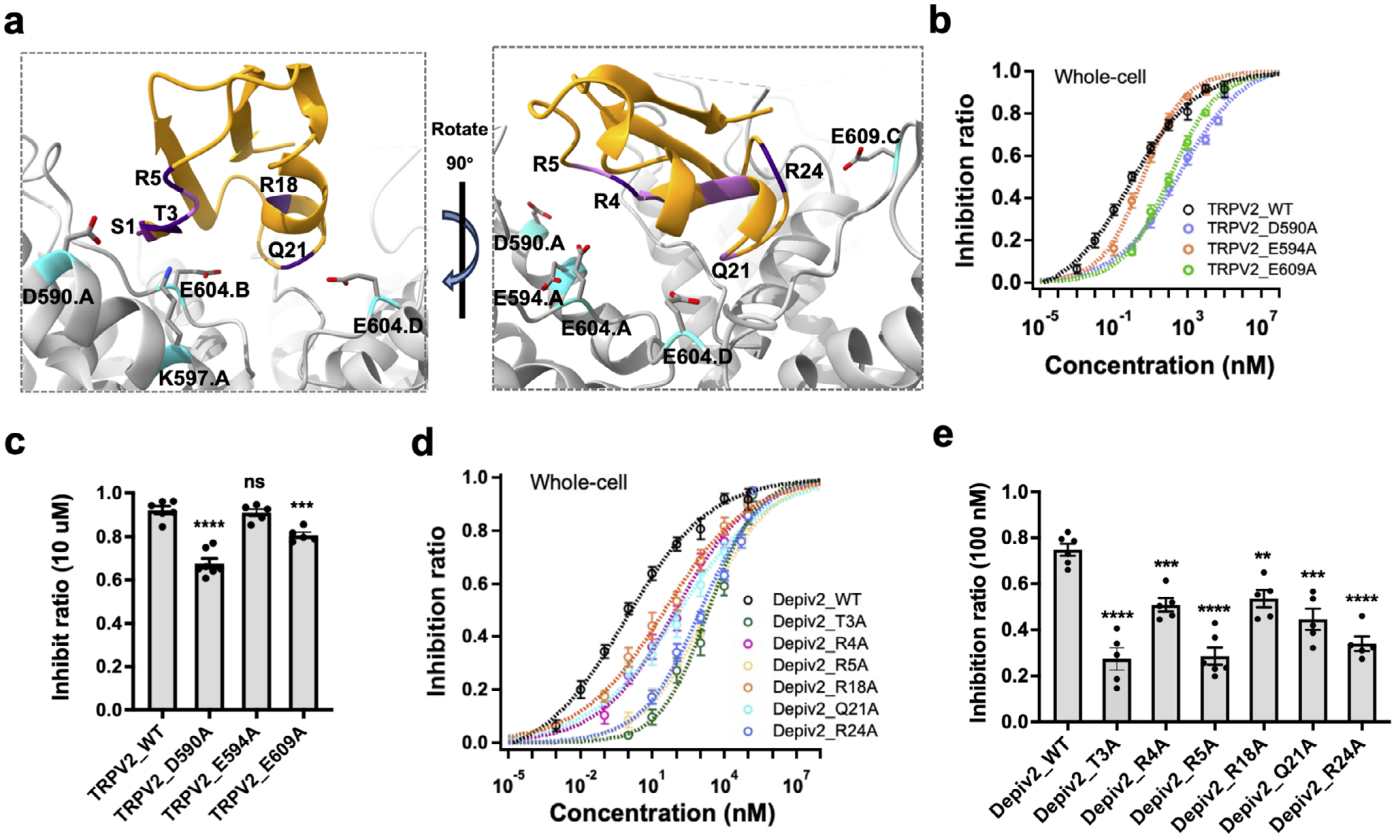
Key residues involved in TRPV2 inhibition by Depiv2. (a) Key amino acids involved in Depiv2‐mediated inhibition of the TRPV2 channel. The right view was generated by rotating and tilting the left view. The TRPV2 channel is shown in light gray, with key residues highlighted in cyan. Depiv2 is shown in orange, with key residues highlighted in different shades of purple. (b) Concentration–response curves of Depiv2 on wild‐type and mutant TRPV2 channels. Data were collected at –80 mV and are presented as mean ± S.E.M.; *n* = 3–10 independent cells for TRPV2_WT, *n* = 7 for TRPV2 D590A, and *n* = 5 for TRPV2_E594A and TRPV2_E609A. (c) Inhibition ratios of Depiv2 on wild‐type and mutant TRPV2 channels at 10 µM. Statistical comparisons were derived from the concentration–response curves in b. Data are presented as mean ± S.E.M.; *n* = 6 independent cells for TRPV2_WT, *n* = 7 for TRPV2 D590A, and *n* = 5 for TRPV2_E594A and TRPV2_E609A. An unpaired two‐tailed Student's *t*‐test was used; ns, not significant; ^***^ *p* < 0.001; ^****^ *p* < 0.0001 vs. TRPV2_WT. (d) Concentration–response curves of wild‐type and mutant Depiv2 peptides on the TRPV2 channel. Data were collected at –80 mV and are presented as mean ± S.E.M.; *n* = 3–10 independent cells for Depiv2_WT, *n* = 5 for Depiv2_T3A, Depiv2_R4A, Depiv2_R18A, Depiv2_Q21A and Depiv2_R24A, and *n* = 6 for Depiv2_R5A. (e) Inhibition ratios of wild‐type and mutant Depiv2 peptides on the TRPV2 channel at 100 nM. Statistical comparisons were derived from the concentration–response curves in d. Data are presented as mean ± S.E.M.; *n* = 5 for Depiv2_T3A, Depiv2_R4A, Depiv2_R18A, Depiv2_Q21A and Depiv2_R24A, and *n* = 6 for Depiv2_WT and Depiv2_R5A. Statistical significance was assessed using an unpaired two‐tailed Student's *t*‐test; ^**^ *p* < 0.01; ^***^ *p* < 0.001; ^****^ *p* < 0.0001 vs. Depiv2_WT.

To validate the binding configuration of Depiv2 and define its mechanism of TRPV2 inhibition in live cells, we performed systematic mutagenesis guided by the cryo‐EM structure. Residues lining the extracellular pore domain of TRPV2 were substituted (D590A, E594A, K597A, E604A and E609A; Figure 4a and Table S2), and the inhibitory effects of Depiv2 were assessed by whole‐cell electrophysiology. Relative to wild‑type TRPV2, expression of the D590A and E609A mutants at the plasma membrane was slightly reduced (Figure S4a,b). Meanwhile, Depiv2 exhibited significantly right‑shifted concentration–response curves for these mutants (Figure 4b), with reduced inhibition observed at 10 µM (Figure 4c), whereas mutation of E594 exhibited minimal effect. In contrast, K597A and E604A abolished channel activation by 2‐APB, precluding functional assessment of Depiv2 inhibition. These results identify D590 and E609 as key residues for Depiv2 binding.

Complementary alanine substitutions were introduced into Depiv2 peptide (T3A, R4A, R5A, R18A, Q21A and R24A; Figure 4a). All mutant peptides exhibited pronounced right‐shifts in their concentration–response curves compared to Depiv2_WT (Figure 4d), accompanied by significantly reduced inhibition at 100 nM (Figure 4e). Together, these data indicate that multiple residues, particularly the positively charged arginine residues, are critical in the binding of Depiv2 to the TRPV2 channel.

Integration of structural and mutational analyses supports a multivalent interaction model in which Depiv2 engages the extracellular pore through electrostatic and hydrogen‐bonding interactions. Specifically, R4 and R5 are positioned to interact with E604 and D590, respectively, while R18 and R24 likely coordinate E604 and E609 across adjacent subunits. Additional contributions from T3 and Q21 further stabilize the binding interface, collectively forming a distributed interaction network across the extracellular pore domain (Figure 4a).

These findings support a mechanism in which Depiv2 inhibits TRPV2 predominantly through negative allosteric modulation. Binding of Depiv2 to the extracellular pore domain stabilizes conformational rearrangements within the pore domain, including constriction of the S6 bundle crossing, thereby favoring a closed, non‐conductive state. This mechanism is consistent with the marked reduction in channel open probability observed in single‐channel recordings (Figure 1j,n). In parallel, the modest narrowing of the selectivity filter likely contributes to a secondary reduction in ion permeation, in agreement with the observed decrease in single‐channel conductance (Figure 1k,o).

### 2.5 Depiv2 Alleviates Cardiac Hypertrophy in Vitro and in Vivo

TRPV2 serves as a stretch‐activated channel with a critical role in mediating cardiomyocyte hypertrophy in response to increased afterload. Previous studies have shown that genetic ablation of TRPV2 significantly reduces left ventricular hypertrophy in response to pressure overload induced by TAC [16]. Based on these findings, we tested whether Depiv2, the selective inhibitor of TRPV2, could alleviate pathological cardiac hypertrophy.

**FIGURE 5:**
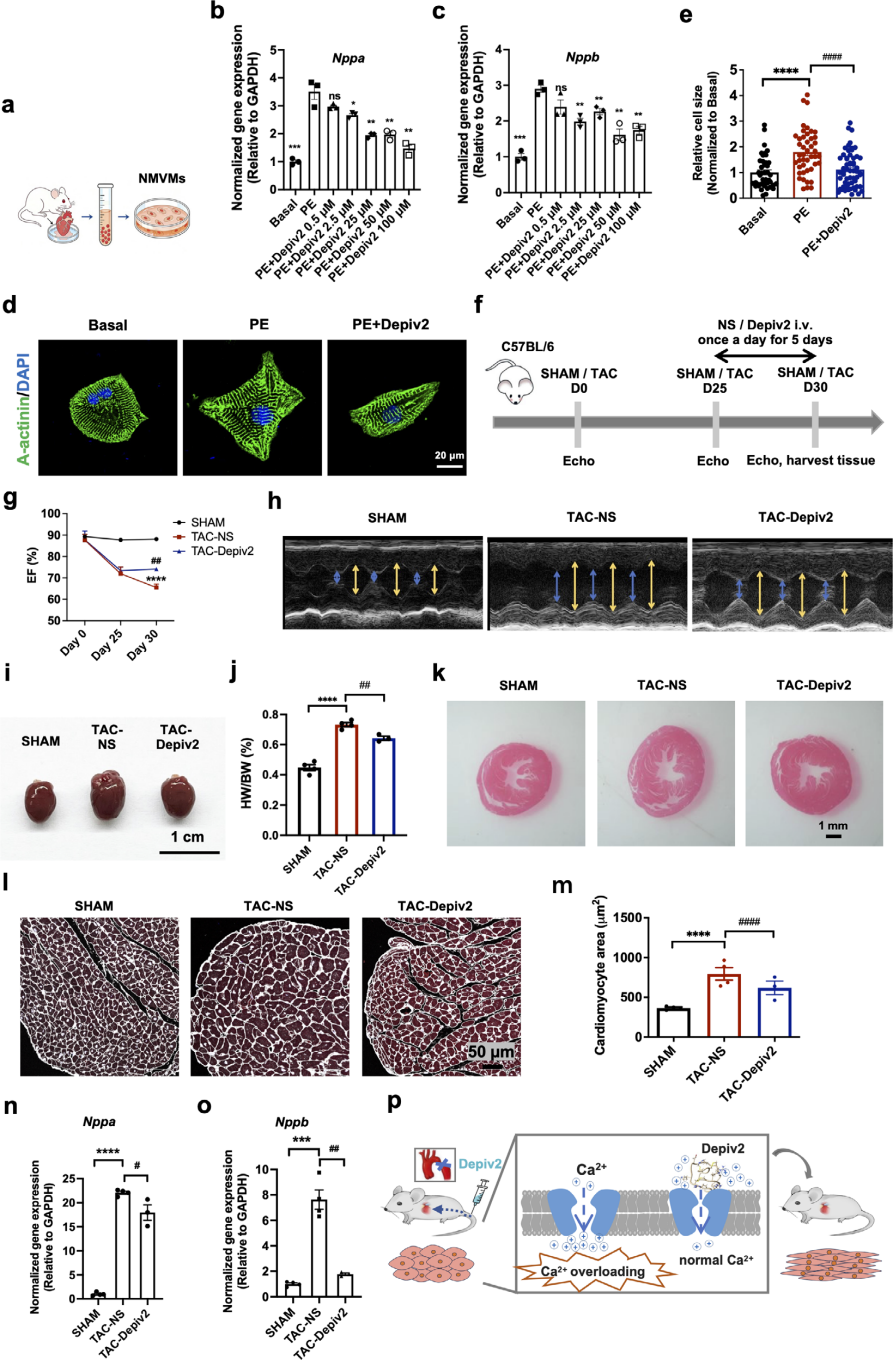
Depiv2 alleviates cardiomyocyte hypertrophy in vitro and TAC‑induced cardiac hypertrophy in mice. (a) Schematic diagram of neonatal mouse ventricular myocyte (NMVM) isolation. (b,c) qRT–PCR analysis of *Nppa* and *Nppb* expression in basal NMVMs, phenylephrine (PE)‐induced NMVMs, and PE‐induced NMVMs treated with Depiv2 (0.5–100 µM). *n* = 3 per group. Data are presented as mean ± S.E.M.; an unpaired two‐tailed Student's *t*‐test was used to compare each group with the PE‐induced NMVM group; ns, not significant; ^*^ *p* < 0.05, ^**^ *p* < 0.01, ^***^ *p* < 0.001. (d,e) Representative images and quantification of cell size in basal NMVMs, PE‐induced NMVMs, and PE‐induced NMVMs treated with Depiv2 (2.5 µM). *n* = 42–58 cells per group. Data are presented as mean ± S.E.M.; an unpaired two‐tailed Student's *t*‐test was used to compare each group with the PE‐induced NMVM group; ^****^ *p* < 0.0001 vs. basal; #### *p* < 0.0001 vs. PE. (f) Schematic depiction of the experimental procedure for TAC‐induced cardiac hypertrophy and Depiv2 treatment. Experiments were performed in adult male C57BL/6 mice (8–10 weeks old). On day 0, TAC or sham surgery was performed. On day 25, echocardiography confirmed successful model establishment, after which TAC mice were divided into two groups and administered either normal saline (NS) or Depiv2 (10 mg/kg) via daily tail vein injection for five consecutive days. On day 30, mice were euthanized and hearts were collected. (g) Ejection fraction (EF) in sham‐operated (SHAM), TAC‐NS and TAC‐Depiv2 mice on days 25 and 30 post‐surgery. Data are presented as mean ± S.E.M.; *n* = 4 mice for SHAM and TAC‐NS, and *n* = 3 mice for TAC‐Depiv2. Statistical significance was assessed using a two‐tailed unpaired Student's *t*‐test; ^****^ *p* < 0.0001 vs. SHAM; ## *p* < 0.01 vs. TAC‐NS. (h) Representative M‐mode echocardiography images recorded on day 30 post‐surgery. (i) Gross heart morphology on day 30 post‐surgery. Scale bar, 1 cm. (j) Heart weight‐to‐body weight ratio in sham‐operated (SHAM), TAC‐NS and TAC‐Depiv2 mice on day 30 post‐surgery. Data are presented as mean ± S.E.M.; *n* = 4 mice for SHAM and TAC‐NS, and *n* = 3 mice for TAC‐Depiv2. Statistical significance was assessed using a two‐tailed unpaired Student's *t*‐test; ^****^ *p* < 0.0001 vs. SHAM; ## *p* < 0.01 vs. TAC‐NS. (k) Hematoxylin and eosin (H&E) staining of heart sections on day 30 post‐surgery. Scale bar, 1 mm. (l) Representative images of heart sections stained with wheat germ agglutinin (WGA; red) and DAPI (blue). Scale bar, 50 µm. (m) Quantification of cardiomyocyte cross‐sectional area. More than 400 cells from at least three hearts were analyzed per condition. Two‐tailed unpaired Student's *t*‐test; ^****^ *p* < 0.0001 vs. SHAM; #### *p* < 0.0001 vs. TAC‐NS. (n,o) qRT–PCR analysis of *Nppa* (n) and *Nppb* (o) in heart tissues on day 30 post‐surgery. Data are presented as mean ± S.E.M.; *n* = 4 mice for SHAM and TAC‐NS, and *n* = 3 mice for TAC‐Depiv2. Statistical significance was assessed using a two‐tailed unpaired Student's *t*‐test; ^***^ *p* < 0.001, ^****^ *p* < 0.0001 vs. SHAM; # *p* < 0.05, ## *p* < 0.01 vs. TAC‐NS. (p) Schematic diagram illustrating the proposed mechanism by which Depiv2 alleviates cardiac hypertrophy through inhibition of TRPV2 and reduction of calcium overload.

In neonatal mouse ventricular myocytes (NMVMs) (Figure 5a), we induced hypertrophy with phenylephrine (PE) treatment, a commonly used agonist to trigger hypertrophic signaling. Depiv2 was applied at concentrations ranging from 0.5 to 100 µM to assess its effects on hypertrophic gene expression. Quantitative PCR analysis of *Nppa* and *Nppb* mRNA levels revealed that Depiv2 significantly attenuated PE‐induced hypertrophy in a concentration‐dependent manner, with notable effects observed at concentrations as low as 2.5 µM (Figure 5b,c). Immunofluorescence staining for α‐actinin further demonstrated that Depiv2 reduced PE‐induced cardiomyocyte surface area expansion (Figure 5d,e).

To assess the effects of Depiv2 in vivo, we used a TAC model to induce pressure overload‐mediated cardiac hypertrophy in mice. Postoperative echocardiography confirmed successful induction of hypertrophy, and mice were divided into three groups: sham‐operated (SHAM), TAC with normal saline (TAC‐NS), and TAC with Depiv2 treatment (TAC‐Depiv2). Depiv2 (10 mg/kg) was administered once daily via tail vein injection for five consecutive days (Figure 5f).

As expected, TAC surgery induced significant cardiac hypertrophy compared to the SHAM group, as evidenced by a reduction in ejection fraction (EF) (Figure 5g), enlargement of the ventricular chambers (Figure 5h), increased heart size (Figure 5i,k), and a higher heart weight‐to‐body weight ratio (HW/BW) (Figure 5j). Additionally, cardiomyocyte cross‐sectional area was significantly increased (Figure 5l,m), and mRNA levels of hypertrophic markers *Nppa* and *Nppb* were upregulated in the TAC‐NS group (Figure 5n,o).

In contrast, Depiv2 treatment significantly attenuated all of these TAC‐induced changes. Echocardiography revealed that Depiv2 effectively suppressed the decline in EF and improved systolic function compared to the TAC‐NS group (Figure 5g,h). Depiv2 also reduced heart size and the HW/BW ratio (Figure 5i,j). Bright‐field images and hematoxylin and eosin (H&E) staining of heart sections showed that Depiv2 alleviated TAC‐induced cardiac enlargement (Figure 5k). Moreover, Wheat Germ Agglutinin (WGA) staining demonstrated that Depiv2 reduced the increase in cardiomyocyte cross‐sectional area observed in TAC‐induced hypertrophy (Figure 5l,m). Finally, qRT‐PCR analysis of cardiac tissue confirmed that Depiv2 treatment downregulated the elevated levels of *Nppa* and *Nppb* mRNA induced by TAC (Figure 5n,o).

These findings demonstrate that Depiv2 alleviates pathological cardiac hypertrophy in both in vitro and in vivo models, in a manner consistent with TRPV2 inhibition. These results underscore the critical role of TRPV2 as a key mediator of cardiac hypertrophy and highlight Depiv2 as a valuable tool for studying TRPV2 in disease contexts, with potential for future exploration in therapeutic development (Figure 5p).

## 3 Discussion

Our study establishes the extracellular pore of TRPV2 as a targetable allosteric site for selective channel modulation. Although extracellular pore domains are known to contribute to ion permeation and gating [3, 5], they have remained underused as targets for rational ligand design. Here we show that this surface can be targeted by a compact engineered peptide to achieve high potency, strong subtype selectivity, and mechanistically defined inhibition. In this sense, Depiv2 is not simply a TRPV2 inhibitor, but a structurally resolved chemical probe that demonstrates the tractability of an extracellular pore domain for rational peptide modulation. More broadly, this work extends prior observations in TRP channels, including toxin‐ and peptide‐mediated modulation of the TRPV1 extracellular pore domain and state‐selective targeting of the TRPM8 extracellular pore [8, 9], by establishing such extracellular surfaces as deliberate design targets rather than isolated mechanistic features.

Our structural and electrophysiological analyses further indicate that Depiv2 acts through pore remodeling rather than simple physical pore blocking. The cryo‐EM structure shows binding within the extracellular pore domain and stabilization of a closed, non‐conductive pore conformation. Consistent with this architecture, Depiv2 reduces both channel open probability and single‐channel conductance (Figure 1j,k,n,o), indicating coupled effects on gating and ion permeation. We therefore interpret Depiv2 primarily as an extracellular negative allosteric modulator that shifts the pore toward a non‐conductive state, while secondarily restricting permeation through a narrowed conduction pathway. This mechanism reinforces the view that the TRP extracellular pore domain is not merely permissive for ion flow, but an allosterically active structural module that can be selectively biased by ligand binding. This property may be partly attributable to the fact that Depiv2 was designed based on a closed‐state TRPV2 structure. It is conceivable that peptides designed against open‐state conformations might preferentially recognize activated channels and thus exhibit different state dependence, kinetics, or selectivity profiles—potentially acting more as pore blockers than as allosteric modulators. Depiv2 may modulate turret‑mediated open‑state stabilization via allosteric effects on the pore architecture, although this possibility remains speculative and requires direct evaluation by future higher‑resolution structural studies and experimental validation.

To promote electrostatic interactions with the negatively charged extracellular pore of TRPV2, we incorporated seven positively charged residues into Depiv2. Functional characterization revealed voltage‑dependent inhibition, with stronger potency at negative potentials, supported by single‑channel recordings showing that both open probability and unitary conductance were suppressed to slightly lower levels at −80 mV, suggesting a general electrostatic contribution to Depiv2 binding. Voltage‑dependent conformational changes in the channel may further modulate binding site accessibility, contributing to the state dependence of inhibition.

More broadly, our study establishes the extracellular pore of TRPV2 as a designable allosteric site, extending prior observations of toxin‐ and peptide‐mediated modulation of TRP channel outer pores. Natural toxins are products of evolutionary empiricism; their discovery proves that extracellular pore surfaces are targetable by exogenous ligands, but it does not reveal whether the physicochemical features of these dynamic, solvent‐exposed surfaces are sufficiently well‐defined to support rational engineering. Unlike naturally evolved toxins identified through biological screening, Depiv2 was generated through a rational structure‐guided design strategy, highlighting the utility of the OHCA platform for developing peptide modulators against extracellular ion‐channel surfaces. The successful de novo design of Depiv2 bridges this critical gap, transiting the extracellular pore from a targetable domain previously accessible only by chance or evolutionary selection to a designable allosteric site. It demonstrates that the gating machinery of TRP channels can be deliberately sculpted through rational peptide engineering, providing a generalizable framework for targeting extracellular regulatory surfaces of membrane proteins.

Notably, comparison of the designed model with the experimentally determined structure revealed only localized deviations in backbone orientation (Figure S3a–c). These differences likely reflect induced‐fit accommodation within the dynamic pore environment rather than failure of the design logic. The preservation of the core hotspot interactions therefore highlights a key strength of OHCA: it prioritizes energetically dominant contacts that remain robust despite local structural adaptation, improving design reliability at conformationally plastic protein interfaces.

Depiv2 also provides a useful tool for interrogating TRPV2 biology. In the cardiovascular setting, selective extracellular inhibition of TRPV2 was sufficient to blunt pathological remodeling in cultured cardiomyocytes and in a pressure‐overload mouse model, supporting the view that TRPV2 can be productively perturbed to intervene in pathological processes [18]. Although Depiv2 exhibited nanomolar potency in electrophysiological assays, its in vivo efficacy required considerably higher doses—a common limitation of peptide therapeutics, likely attributable to tissue distribution, plasma protein binding, enzymatic degradation, and systemic clearance. As its pharmacokinetic profile remains uncharacterized, the dose–exposure relationship is currently undefined. Future assessment of serum stability, biodistribution, and half‑life will be critical. Therefore, further optimization will be required if Depiv2 is to advance beyond a probe scaffold, and the extent to which this strategy generalizes across other ion channels remains to be defined. Even so, our findings provide a framework for targeting extracellular regulatory surfaces of TRP channels and suggest that extracellular pore domains may represent a broader class of chemically accessible, functionally decisive interfaces for peptide‐based modulation of membrane proteins.

## 4 Experimental Section

### 4.1 Chemicals

Capsaicin was purchased from MedChemExpress (MCE). 2‐APB, GSK1016790A, ADPR, AITC, menthol and Cannabidiol were purchased from Sigma‐Aldrich. Probenecid was purchased from GlpBio.

### 4.2 Peptide Design

The de novo design of Depiv2 was performed using the optimized hotspot‐centric approach (OHCA) as previously described [36], which has been successfully applied to the design of peptidic modulators targeting TRP channels, including TRPV1 [10] and TRPM8 [9].

The structure of mouse TRPV2 (PDB ID: 7XEO) was used as the template for peptide design. All natural amino acids were computationally docked into the extracellular pore domain of TRPV2 channel using Rosetta [37]. Among all amino acids tested, arginine exhibited the most favorable interaction energy and was therefore selected as the primary hotspot for subsequent design. Using RosettaScripts [38], an inverse rotamer library for arginine side chains was generated. Candidate protein scaffolds were then selected from the Protein Data Bank (PDB) according to the following criteria: (1) absence of DNA, RNA, or disulfide bonds; (2) monomeric proteins containing a single polypeptide chain; (3) fewer than 40 amino acid residues; (4) absence of bound ligands; and (5) sequence redundancy reduced to 70% identity.

These protein scaffolds were further cleaned and pre‐packed using Rosetta. The scaffold library was then docked to the extracellular pore domain of TRPV2 using PatchDock [39], and scaffolds with complementary shapes were selected. Arginine residues were subsequently introduced into these scaffolds as interaction hotspots. To account for structural perturbations introduced by arginine incorporation, the protein–protein interfaces were redesigned using RosettaScripts, followed by a second round of docking and screening to eliminate poorly binding candidates. We computationally generated 8430 candidates, followed by screening with the criteria of a ddG value lower than −20 Rosetta Energy Units (REU), a SASA greater than 500 Å^2^, and a shape complementarity score greater than 0.6. The design with the most favorable ddG and total score was selected as the final candidate and further subjected to affinity maturation using Rosetta. Among the passing 325 designs, we then chose the 10 designs with the lowest (most favorable) ddG values (binding energy) for visual inspection. All structural models were visualized using UCSF Chimera (version 1.12) [40].

### 4.3 Peptide Synthesis, Purification, and Characterization

Linear Depiv2 and its mutant variants were synthesized by GL Biochem (Shanghai, China) with >95% purity. The peptide (0.2 mg/mL) was oxidized overnight at 28°C in a buffer containing 0.1 M NaCl, 0.1 M Tris, 5 mM reduced glutathione, and 0.5 mM oxidized glutathione (pH 8). The oxidized peptides were purified using an NGC system (Bio‐Rad, USA) equipped with a C18 reverse‐phase column (XBridge BEH, 5 µm), and elution was monitored by UV absorbance at 280 nm, because Depiv2 contains aromatic residues that exhibit strong and specific absorbance at 280 nm. Fractions containing the target peptides were collected, lyophilized, and confirmed by MALDI–TOF or electrospray ionization mass spectrometry (ESI–MS).

### 4.4 Cell Culture and Transient Transfection for Electrophysiology

HEK293T cells, purchased from and authenticated by the American Type Culture Collection (ATCC), were cultured in high‐glucose Dulbecco's modified Eagle's medium (DMEM; Gibco, USA) supplemented with 10% fetal bovine serum (FBS) and 1% penicillin–streptomycin at 37°C in a 5% CO_2_ atmosphere. Cells were seeded in 12‐well plates 8–12 h before transfection. Transient transfection was performed using 3 µL of Lipofectamine 3000 (Thermo Fisher Scientific) and 1 µg of plasmid DNA per well. Electrophysiological recordings were conducted 20–30 h after transfection, and single‐channel recordings were performed 12–14 h after transfection.

Plasmids encoding mouse TRPV1, TRPV2, TRPV3, TRPV4, TRPM4 (TRPM4‐K1045A mutant to eliminate PIP_2_ dependence), and TRPM8 were fused to yellow or green fluorescent proteins to identify transfected cells. Plasmids encoding human TRPM2, TRPA1, Nav1.5, Nav1.7, and hERG were co‐transfected with a GFP‐encoding plasmid (target plasmid:GFP, 10:1). Human TRPV2 was expressed from a dual‑promoter vector containing a separate YFP/GFP marker cassette for identification of transfected cells. Site‐directed mutagenesis of mTRPV2 was performed using the Fast Mutagenesis Kit (Takara Bio), and all mutations were confirmed by DNA sequencing.

### 4.5 Electrophysiology

Patch‐clamp recordings were performed using a HEKA EPC10 amplifier controlled by PatchMaster software (HEKA, Germany). Patch pipettes were fabricated from borosilicate glass and fire‐polished to a resistance of 3–6 MΩ using a P‐97 puller (Sutter, USA). Except for sodium channels and hERG channels, whole‐cell and single‐channel recordings were conducted at ±80 mV. Each sweep consisted of 50 ms at 0 mV, 200 ms at 80 mV, 200 ms at −80 mV, and 50 ms at 0 mV. Currents were sampled at 50 kHz and filtered at 2.9 kHz, with a sweep interval of 1 s. Unless otherwise indicated, recordings were performed at room temperature (25°C).

A gravity‐driven perfusion system (RSC‐200, Bio‐Logic) was used for rapid application of peptides and ligands. Bath and ligand solutions were delivered through separate channels to minimize mixing. The patch pipette was positioned near the outlet of the perfusion tube. Depiv2 stock solutions were freshly prepared and diluted into extracellular solution immediately before use. All peptide applications were performed using the same perfusion system and tubing configuration. To minimize peptide adsorption, the perfusion lines were thoroughly flushed with peptide‐containing solution before data acquisition. For whole‐cell and single‐channel recordings, Depiv2 was applied by continuous perfusion after 2‐APB‐evoked currents reached a steady state.

For TRPV1, TRPV2, TRPV3, TRPV4, TRPM8, and TRPA1 channels, both bath and pipette solutions contained 130 mM NaCl, 0.2 mM EDTA, and 3 mM HEPES (pH 7.2–7.4, adjusted with NaOH). For TRPM4, the bath solution contained 130 mM NaCl and 3 mM HEPES (pH 7.2), while the pipette solution additionally contained 0.5 mM CaCl_2_. For TRPM2, the bath solution contained 147 mM NaCl, 2 mM KCl, 1 mM MgCl_2_, 10 mM HEPES, 2 mM CaCl_2_, and 13 mM glucose (pH 7.2), and the pipette solution contained 147 mM NaCl, 1 mM MgCl_2_, and 10 mM HEPES (pH 7.2). For Nav1.5 and Nav1.7, the bath solution contained 140 mM NaCl, 3 mM KCl, 1 mM MgCl_2_, 1 mM CaCl_2_, and 10 mM HEPES (pH 7.2), while the pipette solution contained 140 mM CsF, 10 mM NaCl, 10 mM MgCl_2_, 3 mM KCl, and 1 mM EGTA (pH 7.2). For hERG, the bath solution contained 160 mM NaCl, 2.5 mM KCl, 1 mM MgCl_2_, 2 mM CaCl_2_, 10 mM HEPES, and 8 mM glucose (pH 7.4), and the pipette solution contained 150 mM KCl, 5 mM MgCl_2_, and 10 mM HEPES (pH 7.4). The composition of the Ca^2^^+^‑containing bath solution was as follows: 140 mM NaCl, 5 mM KCl, 2 mM MgCl_2_, 2 mM CaCl_2_, and 10 mM HEPES (pH 7.3).

Sodium channel currents were evoked by depolarizing pulses from −80 mV to −10 mV. hERG currents were recorded using a protocol consisting of a holding potential at −80 mV, a depolarizing step to +40 mV for 2 s, followed by repolarization to −60 mV for 2 s before returning to −80 mV.

For experiments conducted at elevated temperatures (37°C and above 40°C), temperature control was achieved by gravity‑driven perfusion of preheated extracellular solution. The bath solution was maintained at the desired temperature using a TC‑324C temperature controller (Warner Instruments), with the temperature continuously monitored via the monitor temp output. A TA‑29 miniature bead thermistor (Harvard Apparatus) was positioned immediately adjacent to the recorded cell to ensure accurate measurement of the local temperature throughout the recordings.

### 4.6 Data Analysis

Data from whole‐cell and single‐channel recordings were analyzed using Igor Pro (version 5.05, WaveMetrics). Concentration–response curves were fitted with the Hill equation to determine IC_50_ values. Single‐channel recordings were performed by alternating voltage steps to +80 mV and −80 mV, each lasting 200 ms. For data analysis, we excluded the transient periods between the two voltage steps. Single‐channel conductance and open probability (*N* *P_o_*) (*N* = 1) were calculated from all‐points histograms and fitted with a double Gaussian function.

The double Gaussian function was defined as follows:

$$f\left(x\right)=y_{0}+A_{0}\exp\left[-{\left(\frac{x-x_{0}}{w_{0}}\right)}^{2}\right]+A_{1}\exp\left[-{\left(\frac{x-x_{1}}{w_{1}}\right)}^{2}\right]$$

where y_0_ is a constant; x_0_ and x_1_ denote the current levels of the closed and open states, respectively; A_0_ and A_1_ represent the corresponding peak amplitudes; and w_0_ and w_1_ indicate the peak widths. The open probability (*NP_o_*) (*N* = 1) and single‐channel conductance were calculated as:

$$NP_{o}=\frac{A_{1}w_{1}}{A_{0}w_{0}+A_{1}w_{1}}$$

$$\text{Single-channel}\;\text{conductance}=\frac{x_{1}-x_{0}}{V}\left(V=80\;\mathrm{mV}\right)$$

### 4.7 Fluorescence Imaging of HEK293T Cells

To visualize the expression patterns of wild‐type (WT) and mutant TRPV2 in HEK293T cells, plasmids encoding YFP‐tagged WT or mutant TRPV2 were transiently transfected into HEK293T cells using Lipofectamine 3000 (Life Technologies). Cells were seeded in 12‐well plates and transfected at ∼70% confluency with 1 µg plasmid DNA per well. At 24 h post‐transfection, cells were dissociated with trypsin, resuspended in complete culture medium, and replated onto glass‐bottom dishes under standardized conditions to ensure comparable cell density across all groups. Cells were allowed to adhere for 4–6 h prior to imaging. Images were acquired using a fast high‐resolution laser scanning confocal microscope (STELLARIS 8 FLIM, Leica Microsystems, Germany). YFP fluorescence was excited using a 514 nm laser line, and emission was collected within the appropriate spectral window. Bright‐field images were obtained using transmitted light. All images were acquired under identical settings and analyzed using ImageJ software (National Institutes of Health).

### 4.8 Protein Expression and Purification

The full‐length mouse *TRPV2* gene (NCBI Gene ID: 22368), containing an N‐terminal Flag and Strep‐tag II, was cloned into the pEG‐BM vector. TRPV2 was heterologously expressed in HEK293F GnTI^−^ suspension cells (Life Technologies) using the BacMam system (Thermo Fisher Scientific). P3 baculovirus generated in Sf9 cells was used to transduce HEK293F GnTI^−^ cells at a ratio of 1:10 (virus:cells, v/v) when the cell density reached ∼4 × 10^6^ cells/mL. Sodium butyrate (10 mM) was added 12 h post‐transduction, and cells were cultured at 30°C to enhance protein expression.

Cells were harvested by centrifugation at 3000 × g, flash‐frozen in liquid nitrogen, and stored at −80°C. Cell pellets were resuspended in lysis buffer A (20 mM HEPES, pH 7.4, 150 mM NaCl) supplemented with protease inhibitors (2 µg/mL DNase I, 0.5 µg/mL pepstatin, 2 µg/mL leupeptin, 1 µg/mL aprotinin, and 100 µM phenylmethylsulfonyl fluoride) and lysed by sonication on ice.

Membrane proteins were solubilized with 1% (w/v) n‐dodecyl‐β‐D‐maltopyranoside (DDM; Anatrace) for 3 h at 4°C. The supernatant was collected after ultracentrifugation at 48,000 × g for 1 h and incubated with Strep‐Tactin Sepharose resin (IBA) at 4°C. The resin was loaded onto a gravity column, washed with 20 column volumes of buffer B (buffer A supplemented with 0.05% DDM), and the protein was eluted with buffer C (buffer A containing 0.05% DDM and 10 mM desthiobiotin; Sigma).

### 4.9 Reconstitution of TRPV2 Into Nanodisc

Membrane scaffold protein MSP1E3D1 was expressed and purified from Escherichia coli. POPC lipids (Avanti Polar Lipids), dissolved in chloroform, were dried under nitrogen and resuspended in buffer containing 3% (w/v) methyl‐β‐cyclodextrin (MβCD) to a final concentration of 15 mM. Purified TRPV2 was concentrated and mixed with POPC and MSP1E3D1 at a molar ratio of 1:10:2 (TRPV2:POPC:MSP1E3D1). The mixture was incubated at 4°C for 1 h, followed by the addition of Bio‐Beads SM2 adsorbents (Bio‐Rad) in two batches over 16 h to remove detergents. Reconstituted nanodiscs were separated from empty nanodiscs by size‐exclusion chromatography using a Superose 6 Increase 10/300 GL column (Cytiva) pre‐equilibrated with buffer D (buffer A supplemented with 2 mM MgCl_2_). All buffers used during purification were supplemented with 2 mM MβCD unless otherwise stated. Peak fractions corresponding to TRPV2‐containing nanodiscs were collected and concentrated to ∼2 mg/mL for subsequent cryo‐EM analysis.

### 4.10 Cryo‑Electron Microscopy Sample Preparation and Data Acquisition

Cryo‐EM data were collected at the Center of Cryo‐Electron Microscopy, Zhejiang University. For the mTRPV2–Depiv2 complex, 2‐APB was added to a final concentration of 1 mM. Depiv2 was mixed with TRPV2 at a molar ratio of 1:10 (TRPV2:Depiv2) and incubated on ice for 30 min, followed by centrifugation at 14 000 rpm for 10 min. Cryo‐EM grids were prepared by applying 3 µL of sample to glow‐discharged holey carbon grids (Quantifoil Cu R1.2/1.3, 300 mesh). Grids were blotted for 3.5 s at 100% humidity and 4°C, and plunge‐frozen in liquid ethane using a Vitrobot Mark IV (FEI). Data were acquired on a Titan Krios microscope (FEI) operated at 300 kV, equipped with a Selectris energy filter and a Falcon 4 detector. Automated data collection was performed using EPU software. Images were recorded at a nominal magnification of ×140 000, corresponding to a pixel size of 0.93 Å. The defocus range was set from −0.8 to −1.6 µm. Movies were dose‐fractionated into 40 frames at a dose rate of 7.49 e^−^ pixel^−^^1^ s^−^^1^, with a total exposure time of 6 s, resulting in a total dose of ∼52 e^−^ Å^−^^2^.

### 4.11 Image Processing

Movie stacks were motion‐corrected, and contrast transfer function (CTF) parameters were estimated using cryoSPARC (v.3.3.2) [41]. A total of 786,494 particles were automatically picked from 2402 micrographs and extracted in cryoSPARC. Particles were subjected to 2D classification, followed by ab initio reconstruction with C1 symmetry. Heterogeneous refinement was performed using multiple ab initio volumes as initial references to separate distinct conformational states.

One 3D class showing clear structural features was selected for further analysis. After non‐uniform refinement with C1 symmetry, 11 575 particles yielded a reconstruction at an overall resolution of 3.6 Å. Resolution was estimated using the gold‐standard Fourier shell correlation (FSC) criterion (0.143) with a soft mask applied around the protein density. Local resolution was estimated using cryoSPARC.

### 4.12 Model Building, Refinement and Validation

An initial atomic model of mTRPV2–Depiv2 was generated by fitting the mouse TRPV2 structure (PDB: 7XEO) into the 3.6 Å resolution map using Coot. The model was manually adjusted in Coot, with amino acid assignment guided by the densities of bulky residues, including phenylalanine, tryptophan, tyrosine, and arginine.

The model was refined against the final map using real‐space refinement in PHENIX, with secondary structure restraints and non‐crystallographic symmetry applied. The final model comprises residues 67–411, 423–557, and 582–714.

Model geometry was evaluated using MolProbity. Pore radii were calculated using the HOLE program [42]. Structural figures were prepared using PyMOL [43], UCSF Chimera [40], and Coot [44].

### 4.13 Molecular Dynamics Simulations

Molecular dynamics (MD) simulations were performed as previously described [23] using NAMD (version 3.0) [45] with the CHARMM36m force field. The mTRPV2–Depiv2 structural model was embedded into a POPC lipid bilayer using the Membrane Builder module of the CHARMM‐GUI webserver. The system was solvated in a water box with a buffer distance of 15 Å and neutralized with counterions (Na^+^ or Cl^−^), with an ionic concentration of 150 mM NaCl.

Following energy minimization, six equilibration steps were carried out with gradually reduced harmonic restraints applied to the protein, lipids, water, and ions. Production simulations were performed in the NPT ensemble at 30°C and 1 bar using the Langevin thermostat and the Nosé–Hoover Langevin piston method. Long‐range electrostatics were treated using the particle mesh Ewald (PME) method.

Four independent trajectories of 100 ns each were generated from the same initial structure with different random initial velocities to assess reproducibility and conformational sampling. Trajectories were saved every 100 ps for analysis. Root mean square deviation (RMSD) of Cα atoms was calculated relative to the starting structure after least‐squares fitting. Hydrogen bonds were defined using a distance cutoff of 3 Å and an angle cutoff of 30°. All analyses and visualizations were performed using VMD [46].

The distance between Depiv2 and the TRPV2 pore center was calculated from all‑atom molecular dynamics trajectories. Coordinates were saved every 50 000 steps (0.1 ns per frame), and four independent 100 ns production runs were analyzed for each system. The center of mass (COM) of Depiv2 was computed using all non‑hydrogen atoms. The pore center was defined as the geometric center of the Cα atoms of Glu604 from the four TRPV2 subunits. For each trajectory frame, the Euclidean distance between the Depiv2 COM and the pore center was calculated. All analyses were performed using MDAnalysis 2.x and NumPy.

### 4.14 Animals

Adult male C57BL/6 mice (8–10 weeks old) were group‐housed under controlled conditions (22°C–24°C, 30%–70% relative humidity) with a 12 h light/dark cycle and free access to food and water. Mice were randomly assigned to treatment or vehicle groups prior to injury. All experimental procedures involving animals were approved by the Institutional Animal Care and Use Committee of Zhejiang Center of Laboratory Animals (Approval No. ZJCLA‑IACUC‑20010573).

### 4.15 Transverse Aortic Constriction Surgery

Minimally invasive transverse aortic constriction (TAC) was performed in C57BL/6 mice as previously described. Mice were anesthetized with 2% isoflurane in 100% oxygen. All surgical procedures were conducted under aseptic conditions. Pre‐ and post‐operative gentamicin (5 mg/kg, intramuscular) was administered, and mice received analgesia according to institutional guidelines. A limited median sternotomy was performed from the suprasternal notch to the level of the second rib under a surgical microscope. The aortic arch was exposed by blunt dissection, and a 27‐gauge blunt needle was placed adjacent to the aorta between the brachiocephalic artery and the left common carotid artery to standardize the degree of constriction. A 6‐0 nylon suture was tied securely around both the aorta and the needle, after which the needle was promptly removed. For sham‐operated controls, the same procedure was performed without ligation of the aorta. The chest was closed with a purse‐string suture, and the skin was closed with interrupted sutures. Mice were allowed to recover on a heating pad and were monitored until fully ambulatory.

### 4.16 Peptide Injection

At 25 days post‐TAC surgery, Depiv2 or vehicle was administered via intravenous tail vein injection once daily for 5 consecutive days. Depiv2 was dissolved in sterile saline and administered at a dose of 10 mg/kg, with an equivalent volume of normal saline (NS) used as the vehicle control. Injection volumes were adjusted according to body weight. Mice were gently restrained using a rodent restrainer during administration.

### 4.17 In Vivo Echocardiography

Echocardiography was performed using a Vevo 2100 ultrasound system (VisualSonics, Canada) equipped with a 30 MHz linear array transducer. Mice were lightly anesthetized with isoflurane (1–2% in oxygen), and body temperature was maintained at ∼37°C using a heating pad with continuous monitoring via a rectal probe. Chest hair was removed using a depilatory cream prior to imaging. Two‐dimensional (2D), M‐mode, and spectral Doppler images were acquired from modified parasternal long‐axis and short‐axis views. M‐mode images at the mid‐papillary level were used to measure systolic and diastolic parameters. Left ventricular (LV) mass was calculated using the area–length method. All images were digitally recorded as cine loops (>300 frames per loop) for offline analysis. Image analysis was performed in a blinded manner.

### 4.18 Neonatal Mouse Ventricular Myocyte Isolation and Culture

Primary neonatal mouse ventricular myocytes (NMVMs) were isolated from neonatal mice (P0–P2) using the Neonatal Heart Dissociation Kit (Miltenyi Biotec, 130‐098‐373) according to the manufacturer's instructions. Briefly, ventricular tissues were enzymatically dissociated to obtain single‐cell suspensions. Cardiomyocytes were enriched by pre‐plating to reduce fibroblast contamination.

NMVMs were cultured in high‐glucose Dulbecco's modified Eagle's medium (DMEM; Gibco, USA) supplemented with 10% fetal bovine serum (FBS; Gibco, USA) and 1% penicillin–streptomycin, and then seeded onto culture dishes for subsequent experiments.

### 4.19 Histological Analysis

Hearts were collected immediately after sacrifice and fixed in 4% paraformaldehyde (PFA; Beyotime) for 24 h at 4°C. Fixed tissues were embedded in paraffin and sectioned at a thickness of 5 µm. Sections were stained with hematoxylin and eosin (H&E; Servicebio) to evaluate cardiac morphology and visualized using a light microscope (Olympus VS200, Japan).

To assess cardiomyocyte cross‐sectional area, heart sections were stained with wheat germ agglutinin (WGA; Sigma‐Aldrich). Images were acquired using a confocal microscope (Nikon A1 Ti, Japan), and cell size was quantified using ImageJ software.

### 4.20 Quantification of Cardiomyocyte Size

For cultured cells, neonatal mouse ventricular myocytes (NMVMs) were immunostained with sarcomeric α‐actinin, and fluorescence images were acquired using a confocal microscope (Leica TCS SP8, Germany). Cell surface area was quantified using ImageJ software.

For heart tissue, paraffin sections were stained with wheat germ agglutinin (WGA), and images were captured using a confocal microscope (Nikon A1 Ti, Japan). Cardiomyocyte cross‐sectional area was measured using ImageJ.

For each condition, more than 400 cardiomyocytes from at least three independent hearts were randomly selected and analyzed. Quantification was performed in a blinded manner.

### 4.21 Real‐Time Quantitative PCR (qPCR)

Total RNA was extracted using TRIzol Reagent (Life Technologies) according to the manufacturer's instructions. cDNA was synthesized using the 4 × EZscript Reverse Transcription Mix II (EZB). Quantitative real‐time PCR was performed using SYBR Green PCR Master Mix (Takara) on a real‐time PCR system. Gene expression levels were normalized to the internal control *GAPDH* and calculated using the 2^−ΔΔC^_t_ method.

### 4.22 Statistical Analysis

All experiments were performed with at least three independent biological replicates. Data are presented as mean ± S.E.M. Statistical analyses were performed using GraphPad Prism 9.0 (GraphPad Software, CA, USA). For comparisons between two groups, a two‐tailed unpaired Student's *t*‐test was used. Statistical significance is indicated as follows: *p* > 0.05 indicates no significance. *, ^**^, ^***^, and ^****^ indicate *p* < 0.05, *p* < 0.01, *p* < 0.001, and *p* < 0.0001, respectively. Exact *n* values and statistical details are provided in the figure legends.

## Author Contributions

F.Y. was responsible for peptide design, structural calculation, and molecular dynamics simulations. A.Z. performed electrophysiological functional experiments, protein expression, purification, sample preparation, and data acquisition and model construction for structure, as well as data analysis and manuscript writing. H.W. performed myocardial cell and animal sectioning, experimental operations, data processing, and manuscript writing. L.X. was responsible for model modification, submission to the PDB database, and revision of draft articles and figures. J.W. participated in structural calculation. X.L. participated in MD analysis. A.A. performed mass spectrometry analysis. F.Y., P.L., and L.X. conceived and supervised the project. All authors participated in data analysis and manuscript preparation.

## Use of Generative AI and AI‐Assisted Technologies in the Writing Process

We acknowledge the use of ChatGPT (OpenAI) for assistance in improving the language and clarity of this manuscript.

## Ethics Statement

All animal procedures were approved by the Institutional Animal Care and Use Committee (IACUC) of Zhejiang Center of Laboratory Animals (Approval Number: ZJCLA‐IACUC‐20010573) and were conducted in accordance with relevant institutional and national guidelines.

## Conflicts of Interest

The authors declare no conflicts of interest.

## Supporting information




**Supporting File 1**: advs77759‐sup‐0001‐SuppMat.docx.

**Supporting File 2**: advs77759‐sup‐0002‐SuppMat.xlsx.

## Data Availability

The atomic coordinates of mTRPV2–Depiv2 have been deposited in the Protein Data Bank (PDB) under accession number 25CT, and the corresponding cryo‐EM density map has been deposited in the Electron Microscopy Data Bank (EMDB) under accession number EMD‐80016. Source data are provided with this paper.
