# Supplementary material for: An Extracellular Pore‑Targeting Peptide Defines a Designable Allosteric Site in TRPV2
Source: Adv Sci (Weinh). 2026 Sep 27:e77759. Online ahead of print. doi: 10.1002/advs.77759 (PMC13616345; doi:10.1002/advs.77759)
Supplement: Supplementary file 1 [file ADVS-9999-e77759-s001.docx]

**Supporting Information**

**An extracellular pore-targeting peptide defines a designable allosteric site in TRPV2**

Aiqin Zhu^1,10^, Hongkun Wang^4,5,10^, Jiawei Wang^1,10^, Xiaolu Liu^6^, Aerziguli Aierken^7,8,9^, Lizhen Xu^2*^, Ping Liang^3,4*^, and Fan Yang^1,2*^

^1^Kidney Disease Center of the First Affiliated Hospital and Department of Biophysics, Zhejiang University School of Medicine, Hangzhou 310003, Zhejiang, China.

^2^Liangzhu Laboratory, Zhejiang University Medical Center, Zhejiang University School of Medicine, Hangzhou 311112, Zhejiang, China.

^3^Key Laboratory of combined Multi-organ Transplantation, Ministry of Public Health, the First Affiliated Hospital, Zhejiang University School of Medicine, Hangzhou 310003, Zhejiang, China.

^4^Institute of Translational Medicine, Zhejiang University, Hangzhou 310029, Zhejiang, China.

^5^Hangzhou Institute of Medicine (HIM), Chinese Academy of Sciences, Hangzhou 310018, Zhejiang, China.

^6^School of Basic Medical Sciences and Forensic Medicine, Hangzhou Medical College, Hangzhou 310059, Zhejiang, China.

^7^Department of Physiology, School of Basic Medical Sciences, Xinjiang Medical University, Urumqi 830017, Xinjiang, China.

^8^Xinjiang Key Laboratory of Molecular Biology for Endemic Diseases, Urumqi 830017, Xinjiang, China.

^9^Scientific Research Platform of Xinjiang Medical University Key Laboratory of Neurophysiology and Clinical Translation, Urumqi 830017, Xinjiang, China.

^10^These authors contributed equally.

^*^Correspondence should be addressed to:

Fan Yang (fanyanga@zju.edu.cn), Ping Liang (pingliang@zju.edu.cn), Lizhen Xu (lzxu777@zju.edu.cn).

Supplementary figure legends

**Figure S1. Structure determination of mTRPV2_Depiv2. a**, Representative size-exclusion chromatography profile of mTRPV2 on a Superose 6 column (GE Healthcare), along with SDS–PAGE analysis of the final sample. **b**, Representative cryo-EM micrograph of mTRPV2–Depiv2. **c**, Flowchart of image processing for mTRPV2–Depiv2 particles. **d**, Cryo-EM density map of mTRPV2–Depiv2 colored by local resolution (Å); side view (left) and top view (right). **e**, Angular distribution of particle orientations used for the final reconstruction. **f**, Gold-standard Fourier shell correlation (FSC) curves calculated using different masks in cryoSPARC. **g**, Representative density maps of the six transmembrane helices (TMs) of mTRPV2–Depiv2. Density corresponding to 2-APB is also shown. Maps are displayed as masks around each TM at a threshold of 0.15 in ChimeraX, low-pass filtered to 3.6 Å, and sharpened with a B factor of −65.9 Å². **h**, Density map of 2-APB in the mTRPV2–Depiv2 complex.

**Figure S2. Design, identification and characterization of Depiv2 and reduced activity of scrambled peptide. a**, Cryo-EM structure of mTRPV2 in the apo state (PDB ID: 7XEO). The extracellular pore domain is highlighted with a green overlay and shown in an enlarged view on the right. **b**, An arginine (brown) stably docks into the extracellular pore domain of TRPV2 as the optimal hotspot. **c**, Depiv2 (colored in purple) binds to the extracellular pore domain of TRPV2, with three pairs of disulfide bonds colored in yellow. **d**, Purification of Depiv2 by reverse-phase chromatography, with the elution peak observed at 38% acetonitrile. **e**, Mass spectrometry analysis showing the molecular weight of linear (left) and folded (right) Depiv2. **f**, Sequence of Depiv2_scrambled. **g**, Depiv2_scrambled inhibited 2-APB–induced TRPV2 currents, with markedly reduced potency. **h**, Concentration–response curves showing a pronounced rightward shift for Depiv2_scrambled (orange) compared with Depiv2 (black), with an IC₅₀ of 5.88 ± 1.01 μM (data were collected at –80 mV and are presented as mean ± S.E.M.; *n* = 6 independent cells). **i**. Representative outside‑out single‑channel recordings showing the entire real‑time recording of 2‑APB and Depiv2 application. Recordings were performed at alternating holding potentials of +80 mV and –80 mV. Each sweep consisted of a 50 ms step to 0 mV, followed by 200 ms steps to +80 mV and –80 mV, and a final 50 ms step to 0 mV. Currents were sampled at 50 kHz and filtered at 2.9 kHz.

**Figure S3. Structural comparison of the designed Depiv2 and the Depiv2 structure determined by three-dimensional reconstruction, along with MD simulation analysis of peptide–pore interactions. a**, Structural model of the designed Depiv2 (purple). The N terminus (N), C terminus (C), and cysteine residues are indicated, with disulfide bonds shown in yellow. **b**, Structural model of Depiv2 determined by cryo-EM (orange). The N terminus (N), C terminus (C), and cysteine residues are indicated, with disulfide bonds shown in yellow. **c**, Structural alignment of the designed Depiv2 and the cryo-EM structure. The overall Cα RMSD between the two models is 5.11 Å. **d**, Ensemble of Depiv2 conformations in the outer pore domain of TRPV2 from MD simulations, showing a stable binding mode. **e**, RMSD of Depiv2 over simulation time for four independent MD trajectories. The RMSD values of all four trajectories stabilized at around 5Å. **f**. Distance between the Depiv2 center of mass and the TRPV2 pore center (defined by Glu604 Cα atoms from the four subunits) over 100 ns MD simulations. Four independent trajectories are shown.

**Figure S4. Confocal imaging results and quantification of membrane localization. a**, Representative fluorescence (top) and bright-field (bottom) images of HEK293T cells expressing yellow fluorescent protein (YFP)-tagged wild-type (WT) and mutant TRPV2. YFP fluorescence was excited using a 514 nm laser line and showed similar distribution patterns in WT- and mutant-expressing cells. Images were analyzed using ImageJ. A line was drawn across each cell to generate fluorescence intensity profiles (red). **b**, Quantification of the plasma membrane (PM)-to-endoplasmic reticulum (ER) fluorescence intensity ratio, reflecting TRPV2 localization at the plasma membrane. Based on the fluorescence images in **a**, the PM-to-ER ratio was calculated as (peak1 + peak4) / (peak2 + peak3), where peaks 1 and 4 correspond to the plasma membrane and peaks 2 and 3 correspond to adjacent ER regions. Data are presented as mean ± S.E.M.; *n* = 3 independent cells per group. A two-tailed unpaired Student’s *t*-test was used for statistical analysis; ns, not significant; *P* < 0.05, *** *P* < 0.001 versus WT.

**Figure S5.** **Validation of mTRPV2-mediated currents and characterization of Depiv2 inhibition. a–c**, Comparison of 1 mM 2‑APB‑evoked currents between blank HEK293T cells and mTRPV2‑expressing HEK293T cells in inside‑out, cell‑attached and outside‑out patch‑clamp configurations. Representative recordings from blank cells (top panels) and mTRPV2‑expressing cells (bottom panels) are shown for each configuration. In the outside‑out recordings, mTRPV2‑expressing cells were recorded within 8–12 h post‑transfection. **d**, Representative concentration‑dependent inhibition of 2‑APB‑evoked mTRPV2 currents by Depiv2 in whole‑cell patch‑clamp recordings. Current traces were obtained at holding potentials of –80 mV and +80 mV. **e–f,** Representative long-term application and repeated pulse stimulation of 1 mM 2‑APB on mTRPV2 currents, minimal desensitization was observed under both conditions. Data were collected from at least 5 independent cells for each condition. **g–h**, Inhibition of mTRPV2 currents by 1 nM Depiv2 applied post‑activation (green box) or pre‑incubated before activation (orange box). **g.** Representative whole‑cell recording from the same cell. **h**. Statistical comparison of inhibition ratios. Pre‑incubation showed significantly higher inhibition (66.09 ± 3.39 %) than post‑activation application (51.25 ± 1.47 %). Data were collected at –80 mV and are presented as mean ± S.E.M.; *n* = 6 independent cells. Statistical significance was assessed using an unpaired two-tailed Student’s *t*-test, ** *P* < 0.01.

**Figure S6. Figure S6. Characterization of Depiv2-mediated inhibition of mouse and human TRPV2 under different activating conditions.** All recordings were performed in whole‑cell configuration at holding potentials of –80 mV and +80 mV. **a**, Representative activation of mTRPV2 by increasing concentrations of Probenecid (0.6, 0.8, 1.0, and 1.2 mM). **b**, Representative concentration-dependent inhibition of Probenecid-evoked mTRPV2 currents by Depiv2. mTRPV2 was activated by Probenecid (1 mM) in the presence of 2 mM extracellular Ca²⁺, followed by application of increasing concentrations of Depiv2. **c**, Activation of mTRPV2 by 1 mM Probenecid in the absence or presence of extracellular Ca²⁺. In the absence of Ca²⁺, Probenecid evoked a rapid and robust current; however, in the presence of 2 mM Ca²⁺, no detectable current was elicited. **d**, Representative activation of mTRPV2 by cannabidiol (CBD, 50 μM) in the absence or presence of 2 mM extracellular Ca²⁺. In the absence of extracellular Ca²⁺, CBD evoked a rapid and robust current, whereas in the presence of 2 mM Ca²⁺, the current developed more slowly and exhibited a markedly reduced amplitude. **e**, Representative concentration-dependent inhibition of CBD-evoked mTRPV2 currents by Depiv2 in the presence of 2 mM extracellular Ca²⁺. Depiv2 was applied at 1 and 1000 nM. **f**, Representative recordings from mock-transfected HEK293T cells under the same conditions as in e. CBD (50 μM) was applied in the presence of 2 mM extracellular Ca²⁺, followed by Depiv2 (1 or 1000 nM). No detectable currents were observed under either condition. **g**, Representative activation of mTRPV2 by 2-APB (1 mM) in the presence of 2 mM extracellular Ca²⁺. **h,** Representative concentration‑dependent inhibition of 2‑APB‑evoked mTRPV2 currents by Depiv2 in the presence of 2 mM extracellular Ca²⁺. **i**, Representative activation of hTRPV2 by CBD (50 μM) in the presence of 2 mM extracellular Ca²⁺. **j**, Representative concentration‑dependent inhibition of CBD‑evoked human TRPV2 currents by Depiv2 in the presence of 2 mM extracellular Ca²⁺. **k**, Concentration–response curves of Depiv2-mediated inhibition of TRPV2 under different activating conditions. The inhibition ratio was calculated by measuring the current amplitude inhibited by Depiv2 at each concentration and normalizing it to the current elicited by the respective agonist. Data were collected at −80 mV and are presented as mean ± S.E.M. The concentration–response curve for mTRPV2 activated by 2-APB (mTRPV2_2APB, black, IC₅₀ = 0.95 ± 0.18 nM) was derived from **Figure 1g**. For mTRPV2 activated by Probenecid (mTRPV2_Probenecid, orange), IC₅₀ = 1.45 ± 0.15 nM, *n* = 3; for mTRPV2 activated by 2-APB in the presence of 2 mM extracellular Ca²⁺ (mTRPV2_Ca²⁺_2APB, green), IC₅₀ = 0.84 ± 0.14 nM, *n* = 4; and for hTRPV2 activated by CBD in the presence of 2 mM extracellular Ca²⁺ (hTRPV2_Ca²⁺_CBD, blue), IC₅₀ = 1.07 ± 0.24 nM, *n* = 3. **l**, Inhibition ratios of TRPV2 by 1 nM Depiv2 under different activating conditions and between mouse and human TRPV2. The inhibition ratios were 51.25 ± 1.47% for mTRPV2_2APB, 43.65 ± 1.87% for mTRPV2_Probenecid, 51.47 ± 5.15% for mTRPV2_Ca²⁺_2APB, 57.14 ± 4.84% for mTRPV2_Ca²⁺_CBD, and 53.22 ± 4.67% for hTRPV2_Ca²⁺_CBD. Data are presented as mean ± S.E.M. **m**, Representative inhibition of 2-APB-evoked mTRPV2 currents by 1 nM Depiv2 under near-physiological conditions (37 °C and 2 mM extracellular Ca²⁺). The temperature trace is shown below the current recording. **n**, Rapid desensitization of 2‑APB‑evoked mTRPV2 currents at temperatures above 40 °C in the presence of 2 mM extracellular Ca²⁺. **o**, Statistical comparison of the inhibition ratios of 1 nM Depiv2 at 25 °C and 37 °C. The inhibition ratios were 51.47 ± 4.81% (*n* = 4) and 50.47 ± 4.12% (*n* = 5), respectively, with no significant difference between the two temperatures (*ns*, unpaired two-tailed Student’s *t*-test). The 25 °C data were derived from the 1 nM Depiv2 condition in **h**. Data are presented as mean ± S.E.M.
